# Effects of prior acute exercise on circulating cytokine concentration responses to a high-fat meal

**DOI:** 10.1002/phy2.40

**Published:** 2013-08-22

**Authors:** Josef Brandauer, Rian Q Landers-Ramos, Nathan T Jenkins, Espen E Spangenburg, James M Hagberg, Steven J Prior

**Affiliations:** 1 Department of Health Sciences, Gettysburg CollegeGettysburg, Pennsylvania, 17325; 2 Department of Kinesiology, School of Public Health, University of MarylandCollege Park, Maryland, 20742; 3 Department of Biomedical Sciences, College of Veterinary Medicine, University of MissouriColumbia, Missouri, 65211; 4 Division of Gerontology and Geriatric Medicine, Department of Medicine, University of Maryland School of MedicineBaltimore, Maryland, 21201; 5 Baltimore Veterans Affairs Geriatric Research, Education and Clinical Center and Research and Development ServiceBaltimore, Maryland, 21201

**Keywords:** Cardiovascular disease, inflammation, lipemia, metabolism

## Abstract

High-fat meal consumption alters the circulating cytokine profile and contributes to cardiometabolic diseases. A prior bout of exercise can ameliorate the triglyceride response to a high-fat meal, but the interactive effects of exercise and high-fat meals on cytokines that mediate cardiometabolic risk are not fully understood. We investigated the effects of prior exercise on the responses of circulating tumor necrosis factor-α (TNF-α), interleukin-6 (IL-6), IL-8, leptin, retinol-binding protein 4 (RBP4), vascular endothelial growth factor (VEGF), basic fibroblast growth factor (bFGF), placental growth factor (PlGF), and soluble fms-like tyrosine kinase-1 (sFlt-1) to a high-fat meal. Ten healthy men were studied before and 4 h after ingestion of a high-fat meal either with or without ∼50 min of endurance exercise at 70% of VO_2_ max on the preceding day. In response to the high-fat meal, lower leptin and higher VEGF, bFGF, IL-6, and IL-8 concentrations were evident (*P* < 0.05 for all). There was no effect of the high-fat meal on PlGF, TNF-α, or RBP4 concentrations. We found lower leptin concentrations with prior exercise (*P* < 0.05) and interactive effects of prior exercise and the high-fat meal on sFlt-1 (*P* < 0.05). The high-fat meal increased IL-6 by 59% without prior exercise and 218% with prior exercise (*P* < 0.05). In conclusion, a prior bout of endurance exercise does not affect all high-fat meal–induced changes in circulating cytokines, but does affect fasting or postprandial concentrations of IL-6, leptin, and sFlt-1. These data may reflect a salutary effect of prior exercise on metabolic responses to a high-fat meal.

## Introduction

Humans in Western societies spend significant amounts of their time in the postprandial state (Sies et al. 2005). The consumption of a high-fat meal elevates circulating triglyceride (TG) concentrations for up to 8 h, impairs endothelial function, and is strongly linked to the development of atherosclerosis and cardiovascular events (Vogel et al. 1997; Tyldum et al. 2009; Wallace et al. 2010; Klop et al. 2012). Inflammatory cytokine concentrations are elevated during postprandial lipemia (Meneses et al. 2011) and the etiology of several cardiometabolic disorders is at least partly rooted in a persistent state of low-grade inflammation (Wellen and Hotamisligil 2005; Calder et al. 2011). Less is known about the responses of angiogenic cytokine concentrations to a high-fat meal, but elevated concentrations of angiogenic growth factors are associated with pathophysiological vascular remodeling in hypertension and atherosclerosis (Lieb et al. 2009; Zachariah et al. 2012). Hence, the investigation of inflammatory and angiogenic responses to a high-fat meal and strategies to ameliorate these responses are of clinical interest.

Inflammatory pathways can be activated by the induction of tumor necrosis factor-α (TNF-α) or elevated blood glucose or lipid levels, particularly if these elevations persist for an extended time period (Hotamisligil et al. 1993; Brownlee 2001). TNF-α is a central regulator of inflammatory processes and induces interleukin-6 and -8 (IL-6 and IL-8) synthesis (Febbraio and Pedersen 2002; Brat et al. 2005). Together, these cytokines share a strong association with the development and progression of cardiovascular disease (CVD) and metabolic diseases (Wellen and Hotamisligil 2005; Zhang 2008; DiDonato et al. 2012) and levels of these cytokines also have been shown to increase following ingestion of a high-fat meal (Meneses et al. 2011). The adipokine leptin can also induce production of IL-6 and other cytokines and may thus contribute to the overall inflammatory state observed in cardiometabolic disease (see Dubey and Hesong 2006 for review) and a high-fat meal. Another adipokine, circulating retinol-binding protein 4 (RBP4), directly contributes to insulin resistance (Yang et al. 2005; Graham et al. 2006) and activates inflammatory pathways in macrophages, in part by increasing secretion of TNF-α and IL-6 (Norseen et al. 2012). The response of RBP4 to a high-fat meal has not been thoroughly investigated, but an increase in RBP4 could help explain previously described cytokine level increases during lipemia. Together, these cytokines may interact to create an inflammatory environment after the consumption of a high-fat meal.

Other circulating cytokines such as vascular endothelial growth factor (VEGF) and its associated family of growth factors are known for their involvement in physiological angiogenesis and vascular maintenance (Lieb et al. 2009), but they may also play a role in the etiology of CVD. VEGF and basic fibroblast growth factor (bFGF) can mediate neovascularization in pathological situations, and circulating concentrations of these cytokines are associated with the presence and severity of CVD (Ripa et al. 2007; Lieb et al. 2009). In particular, elevated VEGF levels contribute to the development of atherosclerotic plaques within the vasa vasorum and intima, thus promoting instability and rupture of advanced plaques over time (Ribatti et al. 2008). Placental growth factor (PlGF) has been shown to promote angiogenesis within atherosclerotic lesions in conjunction with VEGF (Carmeliet et al. 2001), whereas soluble fms-like tyrosine kinase-1 (sFlt-1), an inhibitor of both VEGF and PlGF (Ahmad et al. 2011), may limit angiogenesis (Bainbridge et al. 2002; Kearney et al. 2002). Given the dual physiological and pathological roles of these vascular growth factors, levels of these cytokines must be maintained within relatively narrow limits. In the context of an elevated inflammatory profile during postprandial lipemia, the regulation of angiogenic growth factor concentrations may be of particular importance.

Our group and others have shown that acute endurance exercise performed ∼12–16 h prior to consumption of a high-fat meal can attenuate the increases in TG and systemic oxidative stress otherwise observed following a high-fat meal (Petitt and Cureton 2003; MacEneaney et al. 2009; Jenkins et al. 2011; Gabriel et al. 2012; Klop et al. 2012). An acute exercise bout can ameliorate the lipemic and insulin response to a high-fat meal (see Petitt and Cureton 2003 for review), but it is not clear whether prior exercise, an established anti-inflammatory strategy (Petersen and Pedersen 2005), can prevent a postprandial increase in systemic cytokine levels. Therefore, the goal of this study was to investigate the effects of a high-fat meal with or without a single preceding exercise bout on a number of proinflammatory circulating cytokines. We hypothesized that the consumption of a high-fat meal would increase circulating cytokine concentrations and that prior exercise would attenuate the changes induced by the high-fat meal.

## Methods

### Subjects

Ten healthy adult men aged 18–35 years were recruited to undergo a high-fat meal test with and without a single preceding bout of standardized acute exercise. Subjects were recreationally to highly active and performed at least 4 h/week of aerobic exercise. A screening examination was used to exclude prospective subjects with systolic blood pressure ≥130 mmHg, diastolic blood pressure ≥90 mmHg, serum total cholesterol ≥200 mg/dL, low-density lipoprotein cholesterol ≥130 mg/dL, high-density lipoprotein cholesterol <35 mg/dL, or fasting glucose ≥100 mg/dL (Quest Diagnostics, Baltimore, MD). The University of Maryland College Park Institutional Review Board approved all study procedures. All subjects provided written informed consent. Details on subject characteristics and study methodology have been published previously (Jenkins et al. 2011).

### VO_2_ max testing

Maximal oxygen consumption (VO_2_ max) was determined using a stationary cycling test to exhaustion (Life Cycle, Schiller Park, IL), as described previously (Jenkins et al. 2011). VO_2_ was measured continuously via an automated indirect calorimetry system (Oxycon Pro; Carefusion Respiratory Care, Yorba Linda, CA). All subjects achieved a valid VO_2_ max as indicated by the plateau criteria (≤200 mL/min increase in total VO_2_ with increase in work rate).

### Acute exercise protocol

We chose an exercise protocol which has been shown previously to reliably reduce the lipemic response to a high-fat meal (Petitt and Cureton 2003). Briefly, subjects exercised on a cycle ergometer until an energy consumption of 2.5 MJ (598 kcal) was achieved. The two high-fat meal tests for each subject were conducted 1 week apart, and the order of treatment preceding the high-fat meal tests (prior exercise/no exercise) was balanced.

For the trial with prior exercise, subjects arrived at the laboratory at 1500 h on the day prior to the high-fat meal and performed stationary cycling at ∼70% of VO_2_ max, as previously described (Jenkins et al. 2011). Exercise was stopped when a total energy expenditure of 2.5 MJ (598 kcal) was reached. For the trial with no prior exercise, subjects remained sedentary for the 24-h period prior to the high-fat meal. All subjects were instructed to maintain the same dietary habits for 72 h preceding each high-fat meal test, which was verified by dietary logs.

To minimize the effect of any meal ingested before the high-fat meal test, all study participants consumed a standard meal consisting of commercially available nutrient bars (Zone Perfect, Abbott Nutrition, Columbus, OH) between 1900 and 2000 h on the evening prior to each high-fat meal test. The number of bars eaten by each participant was portioned relative to body mass to provide 0.5 g of carbohydrate and 20.9 kJ per kg body mass.

### High-fat meal tests

Tests began at 0600 h on the morning following each treatment and were carried out as described previously (Weiss et al. 2007). The test meal contained heavy cream, sugar, chocolate syrup, and nonfat powdered milk. Approximately 84% of total calories in the meal were derived from fat (70% of the fat being saturated and 30% monounsaturated fat), ∼3% from protein, and ∼13% from carbohydrate sources (Hotamisligil et al. 1993; Jenkins et al. 2011). The size of the test meal was normalized to each subject's body surface area (386 g [1362 kcal] per 2 m^2^ body surface area). All subjects consumed their test meals within 5 min and the meals were well tolerated. On average, subjects consumed 1310 ± 34.1 kcal (mean ± standard error of mean [SEM]). Blood samples were drawn just before consumption of the meal and hourly for 4 h after completion of the meal.

### Blood assays

Plasma glucose concentration was analyzed using the glucose oxidase method (2300 Stat Plus; YSI Inc., Yellow Springs, OH). Insulin concentrations were assayed via radioimmunoassay (Millipore, St. Charles, MO). The homeostatic model assessment of insulin resistance (HOMA-IR; Turner et al. 1979) was calculated to assess the overall effect of the prior exercise bout on insulin resistance. As reported previously (Jenkins et al. 2011), serum TG concentrations were analyzed using a colorimetric assay (Sigma, St. Louis, MO). Total areas under the curve (AUC) for TG, insulin, and glucose were calculated using the trapezoidal rule.

Plasma concentrations of IL-6, IL-8, TNF-α, VEGF, bFGF, plGF, and the VEGF and plGF inhibitor sFlt-1 were measured in duplicate in samples from the high-fat meal test (0 and 4 h) using multiplex ultrasensitive enzyme-linked immunoassays (MesoScale Discovery, Gaithersburg, MD). Serum leptin concentrations were analyzed by ELISA (R&D Systems, Minneapolis, MN). The average CV for all measurements ranged from 3.3% to 6.6%. RBP4 Western blots were performed using an antibody specific to human RBP4 (Dako, Carpinteria, CA). Each subject's samples for both conditions and all time points were analyzed on the same assay plate or gel.

### Statistical analyses

Data are presented as means ± SEM. We performed 2 × 2 repeated measures analysis of variance (ANOVA) to test for condition (prior exercise/no exercise) and time (fasting/4 h postprandial) effects. Treatment comparisons (e.g., effect of prior exercise on HOMA-IR and insulin, glucose, and TG AUC) were assessed using paired *t*-tests. Statistical significance was accepted at *P* ≤ 0.05. Before any statistical analysis, residuals of dependent variables were assessed for homogeneity of variance and normal distribution. TG AUC data were log 10 transformed prior to statistical analysis and reconverted for visual presentation.

## Results

Ten healthy, recreationally active men took part in this study. Subject characteristics can be found in Table 1. The mean exercise duration for the submaximal exercise trials to reach 2.5 MJ (598 kcal) of energy expenditure was 47 ± 3.1 min with an average work rate of 193 ± 13 Watts.

**Table 1:** Subject characteristics

	Mean ± SEM
Age (yr)	27 ± 1
BMI (kg/m^2^)	24.6 ± 0.7
Body fat (%)	15.1 ± 1.2
Triglycerides (mg/dL)	78 ± 8.1
LDL-C (mg/dL)	93 ± 4
VLDL-C (mg/dL)	16 ± 1.7
HDL-C (mg/dL)	54 ± 3
Cholesterol (mg/dL)	163 ± 6
Glucose (mg/dL)	82 ± 2
SBP (mmHg)	120 ± 3.0
DBP (mmHg)	75 ± 1.8
MAP (mmHg)	90 ± 1.8
VO_2_ max (L/min)	3.8 ± 0.2
VO_2_ max (mL/kg/min)	48.0 ± 2.2

Yr, year; kg, kilogram; BMI, body mass index; m^2^, square meter; mg/dL, milligrams per deciliter; LDL, VLDL, HDL, low-, very low-, high-density lipoprotein; SBP, DBP, MAP, systolic, diastolic, mean arterial blood pressure; mmHg, millimeters of mercury; VO_2_ max, maximal oxygen consumption; L/min, liters per minute; mL/min/kg, milliliters per minute per kilogram body mass; SEM, standard error of the mean.

### Metabolic responses to a high-fat meal with and without prior exercise

**Figure 1:**
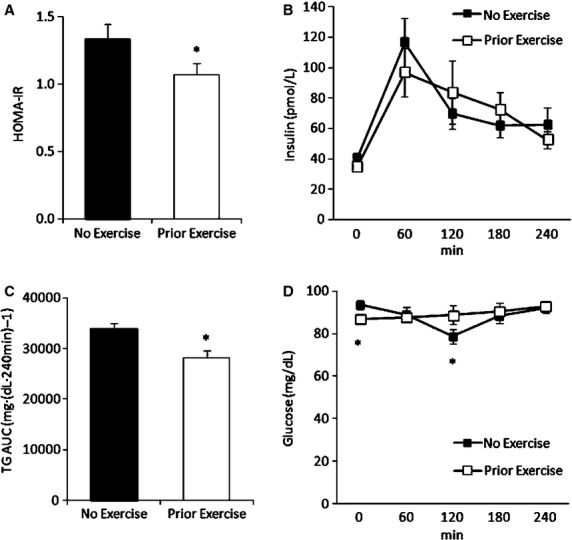
Homeostatic model assessment of insulin resistance (A), and responses of circulating insulin (B), triglyceride (C), and glucose (D) concentrations to a high-fat meal with and without prior exercise. HOMA-IR, homeostatic model assessment of insulin resistance; TG, triglyceride; AUC, area under the curve; pmol, picomoles; min, minutes; mg/dL, milligrams per deciliter. Data are means ± SEM; *n* = 10. *Prior exercise versus no exercise trial (*P* < 0.05).

Plasma insulin, glucose, serum TG, and HOMA-IR data are presented in Figure 1. Compared to the trial without exercise, HOMA-IR was significantly reduced at the start of the high-fat meal with prior exercise (1.33 ± 0.11 vs. 1.07 ± 0.08, respectively, *P* = 0.04; Fig. 1A). This was in large part due to a reduction in fasting glucose levels with prior exercise. Neither the insulin AUC (Fig. 1B) nor glucose AUC (Fig. 1D) was different between the two treatments. As reported previously on these subjects (Jenkins et al. 2011), prior exercise reduced total lipemic load (TG AUC) in response to the high-fat meal by 17% (*P* = 0.03; Fig. 1C).

### Circulating cytokine responses to a high-fat meal with and without prior exercise

**Figure 2:**
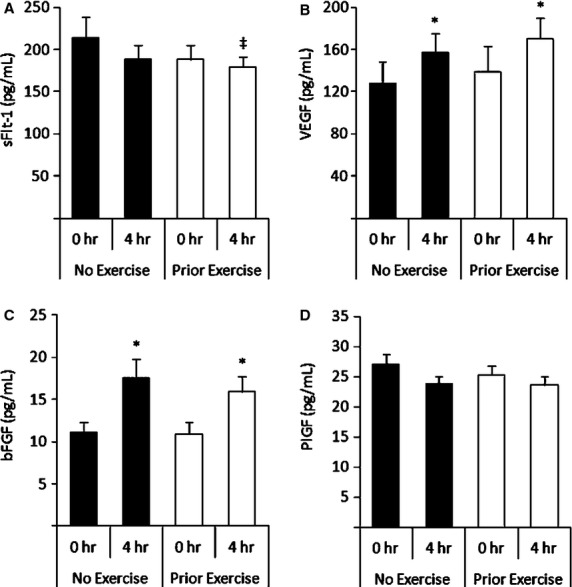
Effects of prior acute exercise on the response of circulating sFlt-1 (A), VEGF (B), bFGF (C), and PlGF (D) to a high-fat meal. sFlt-1, soluble fms-like tyrosine kinase-1; pg/mL, picograms per milliliter; VEGF, vascular endothelial growth factor; bFGF, basic fibroblast growth factor; PlGF, placental growth factor. Data are means ± SEM; *n* = 10. ‡Significant prior exercise and high-fat meal interaction effect (*P* < 0.05), *Significant compared with fasting (0-h) value within condition (*P* < 0.05).

There was a significant interactive effect of treatment (prior exercise/no exercise) and high-fat meal ingestion on the soluble VEGF and PlGF inhibitor sFlt-1 (Fig. 2A, *P* < 0.05). We found significant increases in VEGF concentrations following the ingestion of the high-fat meal both with and without prior exercise (*P* < 0.05; Fig. 2B). Similarly, we detected postprandial increases in bFGF in both conditions (*P* < 0.05; Fig. 2C). Neither prior exercise nor high-fat meal ingestion altered plasma PlGF levels (*P* > 0.05; Fig. 2D).

**Figure 3:**
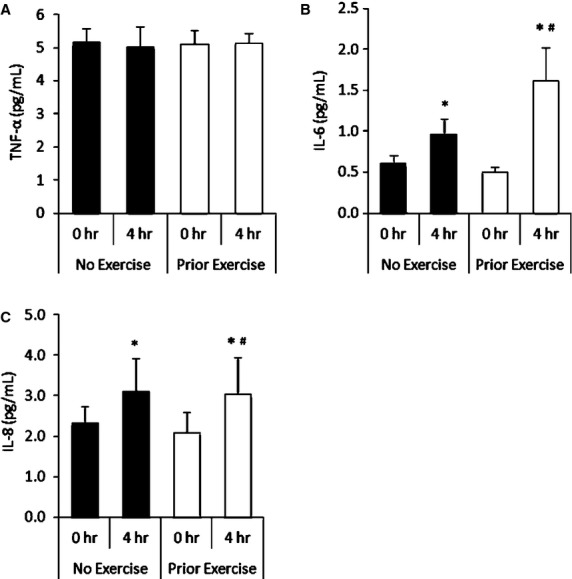
Effects of prior acute exercise on the response of circulating TNF-α (A), IL-6 (B), and IL-8 (C) to a high-fat meal. TNF-α, tumor necrosis factor alpha; pg/mL, picograms/milliliter; IL, interleukin. Data are means ± SEM; *n* = 10. *Significant compared with fasting (0-h) value across conditions (main effect, *P* < 0.05); ^#^Significant compared with fasting (0-h) value within condition (*P* < 0.05).

TNF-α concentrations were unaffected by the high-fat meal with or without prior exercise (main postprandial effect, *P* = 0.97; and main prior exercise effect, *P* = 0.84; Fig. 3A). Fasting IL-6 concentrations were not different between trials, but IL-6 concentrations rose in response to the high-fat meal (main effect, *P* < 0.05; Fig. 3B). This postprandial IL-6 increase was significant with prior exercise (*P* < 0.05) but not with no prior exercise (*P* = 0.16). Similarly, IL-8 concentrations increased in the postprandial state (main effect, *P* > 0.05): The IL-8 increase with prior exercise was statistically significant (*P* < 0.05), whereas the increase with no prior exercise was not (Fig. 3C).

**Figure 4:**
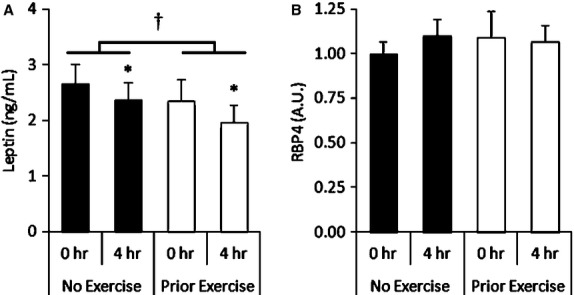
Effects of prior acute exercise on the response of circulating leptin (A) and RBP4 (B) to a high-fat meal. ng/mL, nanograms per milliliter; RBP4, retinol-binding protein 4; A.U., arbitrary units. Data are means ± SEM; *n* = 10. *Significant compared with fasting (0-h) value across conditions (main effect, *P* < 0.05). ^#^Significant compared with fasting (0-h) value within condition (*P* < 0.05); †Significant difference between conditions (main effect of prior exercise *P* < 0.01).

Before and after the high-fat meal, serum leptin concentrations were lower in the trial with prior exercise compared to the trial with no prior exercise (main effect, *P* < 0.01; Fig. 4A). Leptin concentrations decreased in response to the high-fat meal in both trials to similar degrees (main effect, *P* < 0.01). Mean fasting or postprandial serum RBP4 did not change significantly in either condition (prior exercise/no exercise main effect, *P* = 0.63; high-fat meal main effect, *P* = 0.70; Fig. 4B).

## Discussion

The etiology of several cardiometabolic diseases is mechanistically linked to inflammation, but little is known about how exercise, a first-line defense against cardiometabolic disorders, affects postprandial alterations of circulating cytokine concentrations. To address this question, we assessed the effect of prior exercise on circulating cytokine responses to a high-fat meal. The major finding of this study is that acute prior endurance exercise does not attenuate high-fat meal–induced increases in circulating inflammatory cytokines despite improving insulin resistance and reducing postprandial lipemia. We observed postprandial increases in concentrations of proangiogenic (VEGF and bFGF) and inflammatory (IL-6 and IL-8) cytokines both with and without prior exercise. Contrary to our hypothesis, prior exercise may actually result in a larger increase in IL-6 levels after a high-fat meal. Prior exercise decreased fasting concentrations of leptin, and there was an interactive effect of prior exercise and the high-fat meal on sFlt-1 concentrations, wherein prior exercise might reduce fasting sFlt-1 levels or attenuate the response to a high-fat meal.

The consumption of a high-calorie, high-fat meal creates a potent stimulus in which inflammatory cytokines, circulating markers of endothelial stress (Strohacker et al. 2012), excess TG, and oxidized LDL can promote the development of atherosclerotic plaques within the subendothelial space (Vogel et al. 1997; Tyldum et al. 2009; Klop et al. 2012). The increased inflammatory and angiogenic cytokine concentrations we observed after a high-fat meal may represent a component of a proatherogenic postprandial response. We observed improvements in markers of insulin sensitivity when exercise was performed ∼14 h before consumption of the high-fat meal and previously reported reductions in serum TGs and oxidized LDL in these subjects in the prior exercise trials (Jenkins et al. 2011). These findings are in agreement with many other studies demonstrating the positive metabolic (Petitt and Cureton 2003; MacEneaney et al. 2009; Gabriel et al. 2012; Klop et al. 2012) and vascular (Padilla et al. 2006; Tyldum et al. 2009) effects of exercise on the responses following consumption of a high-fat meal. Nevertheless, our present cytokine data did not support our hypothesis that the inflammatory response to a high-fat meal would be attenuated. Thus, our study suggests that the beneficial effects of exercise on postprandial lipid, glucose, and insulin metabolism may be independent of alterations in high-fat meal–induced alterations in circulating cytokine concentrations.

Nappo et al. (2002) reported postprandial increases in IL-6 concentration in subjects with impaired and normal glucose tolerance, although changes in subjects with normal glucose tolerance were relatively small. Strohacker et al. (2012) reported a transient decrease in IL-6 concentration 2 h after consumption of a high-fat meal with prior exercise; however, IL-6 levels were numerically higher at other postprandial time points with prior exercise compared to no prior exercise. Thus, our findings are not necessarily discordant with the present report, but differences in the timing of prior exercise, the high-fat meal, the timing of cytokine measurements, and postexercise caloric replacement make a thorough comparison difficult. Carefully assessing the exact cytokine response time course during postprandial lipemia may be an important contribution to our understanding of the regulation of inflammation. In addition, a recent report showed that consuming a postexercise mixed meal bar after exercise significantly reduced the attenuating effect of prior exercise on postprandial lipemia (Freese et al. 2011). It is possible that replacing the energy deficit of an acute bout of exercise may have blunted salutary effects of prior exercise in our study.

While the source of increased circulating IL levels cannot be ascertained from our study, our results indicate that a prior bout of exercise substantially augments the high-fat meal–induced increase in IL-6 concentrations. Corpeleijn et al. (2005) reported IL-6 release from muscle following a high-fat meal in subjects with impaired glucose tolerance, while Febbraio and Pedersen have suggested that skeletal muscle–derived IL-6 may aid in the maintenance of metabolic homeostasis and have antiinflammatory effects (Febbraio and Pedersen 2002). If increases in IL-6 do contribute to metabolic homeostasis, our observation of elevated postprandial IL-6 concentrations with prior exercise may represent a beneficial effect of exercise after a high-fat meal.

VEGF and bFGF are major mediators of neovascularization in both physiological and pathological situations, and are associated with the presence and severity of CVD (Ripa et al. 2007; Lieb et al. 2009). Thus, it is important for concentrations of these cytokines to be maintained within the appropriate physiological range to ensure proper vascular homeostasis. The postprandial VEGF and bFGF increases we observed after high-fat meal ingestion may indicate an association between postprandial hyperlipidemia and a pathological angiogenic environment that is not ameliorated by prior exercise. PlGF is known to promote angiogenesis within atherosclerotic plaques where it works in synergy with VEGF (Carmeliet et al. 2001). We did not detect any significant change in PlGF concentrations following a high-fat meal with or without prior exercise. It is possible that the acute consumption of a high-fat meal in otherwise healthy, younger men does not affect circulating levels of PlGF, or that any postprandial deflection from fasting concentrations occurs rapidly and transiently. sFlt-1 acts as a soluble inhibitor of both VEGF and PlGF (Ahmad et al. 2011) and has the potential to limit angiogenesis (Bainbridge et al. 2002; Kearney et al. 2002). Our data revealed an interactive effect of prior exercise and the high-fat meal on sFlt-1 concentrations. On one hand, this may be attributed to lower fasting sFlt-1 levels with prior exercise, which would be consistent with a proangiogenic environment in the basal state. However, this interaction could also indicate an attenuation of the high-fat meal–induced decrease in sFlt-1 with prior exercise which would be consistent with a protective effect of exercise. Additional research is needed to clarify the interaction of prior exercise and diet on sFlt-1.

Prior acute exercise significantly reduced basal leptin concentrations. A prolonged effect of acute exercise on basal leptin concentrations has been reported before (for review, see Olive and Miller 2001) even though the total energy expenditure and the exercise duration in our study are lower than previous guidelines for exercise-induced leptin decreases (at least 60 min duration or 800 kcal energy expenditure) (Bouassida et al. 2010). We also observed a significant decrease in leptin in response to the high-fat meal in both conditions. Previous studies have reported early-morning reductions in circulating leptin concentrations so it is possible that the decreases in leptin we observed could at least be partly due to diurnal variations (Nakamura et al. 2008). Regardless of the mechanism responsible for the decrease in leptin following the high-fat meal, prior exercise had no effect on the response of leptin to the high-fat meal (i.e., leptin concentrations decreased similarly in both conditions). Contrary to our findings for leptin, RBP4 concentrations were unaffected by prior exercise or high-fat meal consumption. We hypothesized that RBP4 would increase after meal ingestion and possibly contribute to a rise in circulating TNF-α and IL-6 by activating macrophages as shown by Norseen et al. (2012). The fact that we did not detect increases in either RBP4 or TNF-α despite substantial increases in circulating IL-6 suggests that the diet-induced inflammatory response is complex and relies on various signaling mechanisms.

In conclusion, prior exercise does not appear to ameliorate the effects of a high-fat meal on increases in VEGF, PlGF, or bFGF despite improving insulin sensitivity and reducing lipemia. Reduced fasting leptin levels may indicate a salutary effect of a single acute exercise bout, whereas an augmented increase in IL-6 in the postprandial state may be consistent with improved metabolic control. Alterations in sFlt-1 concentrations with prior exercise could possibly indicate a proangiogenic effect of acute exercise in the basal state or a protective effect in response to a high-fat meal.
